# Photodegradation of Microcystin-LR Using Visible Light-Activated C/N-co-Modified Mesoporous TiO_2_ Photocatalyst

**DOI:** 10.3390/ma12071027

**Published:** 2019-03-28

**Authors:** Tamer M. Khedr, Said M. El-Sheikh, Adel A. Ismail, Ewa Kowalska, Detlef W. Bahnemann

**Affiliations:** 1 Nanomaterials and Nanotechnology Department, Central Metallurgical Research and Development Institute (CMRDI) P.O. Box: 87 Helwan, Cairo 11421, Egypt; selsheikh2001@gmail.com (S.M.E.-S.); aaismail@kisr.edu.kw (A.A.I.); 2 Institute of Technical Chemistry, Photocatalysis and Nanotechnology Research Unit, Leibniz Universität Hannover, Callinstr. 3, D-30167 Hannover, Germany; bahnemann@iftc.uni-hannover.de; 3 Institute for Catalysis, Hokkaido University, N21, W10, Sapporo 001-0021, Japan; kowalska@cat.hokudai.ac.jp; 4 Nanotechnology and Advanced Materials Program, Energy & Building Research Center (EBRC), Kuwait Institute for Scientific Research (KISR), P.O. Box 24885, Safat 13109, Kuwait; 5 Laboratory “Photoactive Nanocomposite Materials” (Director), Saint-Petersburg State University, Ulyanovskaya str. 1, Peterhof, Saint-Petersburg 198504, Russia

**Keywords:** anatase, brookite, C/N-TiO_2_, microcystin-LR, photodegradation, visible light

## Abstract

Microcystin-LR (MC-LR), a potent hepatotoxin produced by the cyanobacteria, is of increasing concern worldwide because of severe and persistent impacts on humans and animals by inhalation and consumption of contaminated waters and food. In this work, MC-LR was removed completely from aqueous solution using visible-light-active C/N-co-modified mesoporous anatase/brookite TiO_2_ photocatalyst. The co-modified TiO_2_ nanoparticles were synthesized by a one-pot hydrothermal process, and then calcined at different temperatures (300, 400, and 500 °C). All the obtained TiO_2_ powders were analyzed by X-ray diffraction (XRD), Raman spectroscopy, transmission electron microscope (TEM), specific surface area (SSA) measurements, ultraviolet-visible diffuse reflectance spectra (UV-vis DRS), X-ray photoelectron spectroscopy (XPS), Fourier transform infrared (FTIR) spectroscopy, and photoluminescence (PL) analysis. It was found that all samples contained mixed-phase TiO_2_ (anatase and brookite), and the content of brookite decreased with an increase in calcination temperature, as well as the specific surface area and the content of non-metal elements. The effects of initial pH value, the TiO_2_ content, and MC-LR concentration on the photocatalytic activity were also studied. It was found that the photocatalytic activity of the obtained TiO_2_ photocatalysts declined with increasing temperature. The complete degradation (100%) of MC-LR (10 mg L^−1^) was observed within 3 h, using as-synthesized co-modified TiO_2_ (0.4 g L^−1^) at pH 4 under visible light. Based on the obtained results, the mechanism of MC-LR degradation has been proposed.

## 1. Introduction

The desire to provide safe potable water has increased due to economic and population growth in recent years. Moreover, global freshwater resources, which might be used for the production of drinkable water, have been even diminished because of water pollution and climate change [1]. Both inorganic and organic compounds of natural, urban and industrial origins are considered as water pollutants. For example, cyanobacteria (commonly known as blue-green algae) are photosynthetic and microscopic organisms existing in aquatic systems, including fresh, brackish and marine water. It has been suggested that an increased content of cyanobacteria in water is caused by eutrophication, climatic changes, available nutrients of agricultural origin and waste disposal in water [1,2,3,4]. They can reproduce to compose harmful algal blooms (HABs) under convenient conditions (high temperatures and nutrient concentrations, and strong sunlight), and some cyanobacteria contain and release toxins as metabolic byproducts and during “cell death” (lysis). These toxic compounds, known as cyanotoxins, are considered a significant threat to human, animals, environment, and even ecosystems due to their high solubility in water, toxicity and chemical stability [1,2,5,6]. There are diverse species of cyanotoxins, detected in the water around the world, but the Environmental Protection Agency (EPA) clearly focused on three of them as being of the biggest threat to the environment: microcystin-LR (MC-LR), cylindrospermopsin (CYN), and anatoxin-a [1,7]. Out of all known cyanobacterial toxins, MC-LR (L for Leucine and R for Arginine) is considered as the most toxic of the microcystins, and the most abundant and harmful in aquatic systems [1,7,8]. MC-LR is a cyclic heptapeptide containing several moieties, including Adda, and other amino acids [7]. The toxicity of MC-LR is large due to the Adda chain, and thus the detoxification requires the removal of the Adda side chain or isomerization from trans to cis. However, the trans-to-cis photoisomerization demands UV irradiation, and thus is impossible under visible light (vis) [7]. Several technologies, including ozonation, chlorination, and phototransformation, have been tested to some extent for removing (and/or detoxifying) MC-LR from water [7,8]. Recently, great attention has been focused on promising green technologies, such as advanced oxidation technologies (AOTs) for removal of cyanotoxins, due to their ability to produce free radicals with strong oxidizing power (mainly hydroxyl radicals) resulting in possible complete degradation (mineralization) of cyanotoxins to CO_2_ and H_2_O. Among AOTs, TiO_2_-based photocatalysis is probably the most widely used for the degradation of emerging pollutants because of promising characteristics, such as non-toxicity for both human and environment, low costs, strong oxidizing ability, photochemical stability, high efficiency, biocompatibility, and facile synthesis with various morphologies [9,10]. Indeed, Feitz et al. reported the decomposition of MC-LR (low concentration) in a natural organic matrix using conventional TiO_2_/UV-based photocatalysis process [11]. Despite the considerable advancement in TiO_2_-based photocatalysis that has been accomplished recently, there are some drawbacks that restrain the photocatalytic performance of TiO_2_. The most serious drawback for TiO_2_ is its large band gap (despite excellent redox ability), which requires UV irradiation (expensive, toxic, and with low content (ca. 4–5%) in the solar spectrum) for photo-excitation, thereby restricting its photocatalytic activity under vis (safe and cheap since natural solar radiation might be used (46% of the sun’s electromagnetic radiation)). In addition, rapid electron–hole recombination (typical for all semiconductors) results in low quantum yields of photocatalytic reactions [7,9,10,12]. Therefore, various strategies, including chemical and structural modifications, have been applied to improve the photocatalytic performance of TiO_2_ [12,13,14,15,16]. It should be noticed that the formation of mixed-phase TiO_2_ nanostructure [9,10], and heterojunctions with other semiconductors [17] facilitate the charge facilitates the charge separation to overcome the electron–hole recombination, and thus enhancing the activity of TiO_2_. The TiO_2_ band gap can be modified by the aggregation state and by differences in particle size, as Reinosa et al. previously demonstrated [18]. Moreover, surface modification and doping (interstitial and substitutional) with metal and non-metal (ions and compounds) have demonstrated to be effective methods for increasing the photocatalytic efficiency of TiO_2_ resulting from enhanced electron–hole separation and appearance of vis absorption [7,8,9,12,13,16]. Recently, research has focused on synthesis of co-modified TiO_2_ because of their higher photocatalytic activity than that of single-modified TiO_2_ [7,8,9,12,13,16]. However, there are only limited studies focusing on the synthesis of non-metal co-modified TiO_2_ for degradation of MC-LR [7]. As far as we know, there is only one report by Liu et al. on the activity of C-N co-modified anatase-TiO_2_ for degradation of MC-LR under visible light irradiation [19]. They prepared C-N co-modified anatase films through a sol-gel method from titanium (IV) isopropoxide (TIP), polyoxyethylene sorbitan monooleate (Tween 80, nonionic surfactant) and anhydrous ethylenediamine (EDA) as Ti, C, and N source, respectively. Unfortunately, titanium alkoxides (e.g., TIP) are very sensitive to moisture, thereby synthesis under inert gas and/or the multi-steps processes (resulting in low yields) must be applied [9,10]. Moreover, incomplete removal (65%) was only achieved for 0.5 mg L^−1^ MC-LR during 5-h vis irradiation (fluorescent lamps with a UV block filter, λ > 420 nm, pH 3). Therefore, in this study, commercially available Ti_2_(SO_4_)_3_ and glycine were used as the TiO_2_-precursor and non-metal ions source, respectively, to prepare C/N co-modified mesoporous A/B TiO_2_ through one-pot surfactant-free hydrothermal approach. Additionally, the as-prepared TiO_2_ powder was further calcined at different temperatures (300, 400, and 500 °C) to investigate the effect of thermal treatment on the properties, and thus photocatalytic activities of TiO_2_ photocatalysts. The as-synthesized and calcined TiO_2_ powders were characterized by advanced techniques, and used for MC-LR degradation using low-cost irradiation source (visible-LED lamp, λ = 420 nm). After 3 h irradiation, the complete removal of MC-LR (C_0_ = 10 mg L^−1^) was achieved using 0.4 g L^−1^ as-prepared C-N co-modified TiO_2_ at pH 4. 

## 2. Materials and Methods

### 2.1. Materials

Titanium(III) sulfate (TS, Ti_2_(SO_4_)_3_, Fisher, Loughborough, Leicestershire, UK, 15%), sodium nitrate (NaNO_3_, Koch-light laboratories Ltd., Haverhill, Suffolk, UK, 98%), glycine (GLY, H_2_N-CH_2_-COOH, Sigma-Aldrich, St. Louis, MO, USA, 99%), sodium hydroxide (NaOH, LobaChemie, Pellets, Mumbai, India, 98%), MC-LR (Cal-Biochem, Nottingham, UK, 99%), and absolute ethanol (CH_3_CH_2_OH, Sigma-Aldrich, Darmstadt, Germany, 99.8%) were used without any further purification. The water used for the experiment was deionized water (DI, 8.2 µ Ω).

### 2.2. Preparation of Photocatalyst

In a typical procedure, two different mixtures were prepared. For the first mixture (A), NaNO_3_ (0.1 µmol L^−1^) was added to TS solution (0.5 µmol L^−1^) and then subjected to gentle stirring for 25 min till the formation of a transparent solution. The second mixture (B) consisting of an aqueous solution of GLY (1 mol L^−1^) and NaOH (2 mol L^−1^) was mixed with a solution (A) dropwise under continuous stirring, and then stirred for 30 min to complete the reaction, resulting in obtaining a milk suspension. Then, the obtained suspension (maintained at 40 mL) was poured into the 100-mL Teflon-lined tube. The sample was treated at 200 °C for 20 h, and then naturally cooled to room temperature, collected, washed several times with ethanol and water, and dried at 60 °C for 12 h. Finally, the as-prepared sample was calcined in a furnace open to air at three temperatures (300, 400, and 500 °C) for 1 h. The as-synthesized and calcined samples were named as CDT-0.00, CDT-300, CDT-400, and CDT-500, respectively.

### 2.3. Characterization of Photocatalyst

The crystal structure of TiO_2_ was analyzed by the X-ray diffraction (XRD, D8, Bruker AXS X-ray diffractometer, Karlsruhe, Germany) in a 2*θ* range from 15 to 70°. Raman spectra were obtained on a Senterra Dispersive Micro-Raman (Bruker, Munich, Germany) under excitation with the 532-nm line of a doubled Nd:YAG laser at an incident power of 10 mW. Transmission electron microscope (TEM, JEOL-JEM-1230, Tokyo, Japan) was used to investigate the particle sizes and morphologies. Brunauer–Emmett–Teller (BET) specific surface areas were estimated based on nitrogen adsorption isotherms at 350 °C using Quanta Chrome Instruments (). All samples were degassed at 180 °C for 12 h before the N_2_ physisorption measurements. The BET specific surface area was estimated using the adsorption data in the relative pressure (P/P_o_). The Barrett–Joyner–Halenda (BJH) pore size distribution was estimated from adsorption data. UV-vis absorption spectra were measured using UV-vis spectrophotometer (UV-2501 PC, Shimadzu, Tokyo, Japan) with an integrating sphere and BaSO_4_ as a reflectance standard. The reflectance data were converted to F(R) values according to the Kubelka–Munk theory. The (F(R) hν)^1/2^ versus energy of the exciting light was plotted to obtain the band-gap energy [10]. The X-ray photoelectron spectroscopy (XPS, Thermo Fisher Scientific, Waltham, MA, USA) measurements were performed to investigate the surface chemical composition. The room temperature FT-IR absorption spectra were recorded using JASCO 3600 spectrometer (Tokyo, Japan), in the spectral range from 400 to 4000 cm^−1^. The PL spectrometer (Shimadzu RF-5301PC, Tokyo, Japan) was used to determine the photoluminescence properties. 

### 2.4. Photocatalytic Experiments

A stock aqueous solution of MC-LR (30 mg L^−1^) was prepared (pH 6.3). Prior the photocatalytic experiments, the standard calibration curve was made for MC-LR concentrations in the range of 5–30 mg L^−1^. The photocatalytic degradation experiments were carried out in a double jacket round quartz reactor with a 50 mL volume. The temperature was maintained at 25 °C by circulation thermostated water around the reactor. Firstly, an aqueous solution of MC-LR (40 mL) was added to a round quartz reactor containing TiO_2_ powder, and then sonicated to obtain a uniform suspension, which was then stirred for 180 min in the dark to achieve the adsorption equilibrium for MC-LR on the catalyst surface before turning the experiments [20]. Thereafter, the suspension was irradiated from the top by a visible-LED lamp (λ_max_ = 420 nm, intensity = 1 mW cm^-2^, height 20 cm). The samples were dragged at different times, and then filtered using a 0.22-μm filter membrane. The residual MC-LR concentration at different durations was adopted using a High-Performance Liquid Chromatography (HPLC, 1260, Agilent, Hamburg, Germany) with a G1311C-1260 Quat pump and a G1365D-1260 MWD UV detector (Hamburg, Germany), set at 238 nm with a C18 column (100 mm Long × 4.6 mm i.d., 3.5 µm particles) and using the method reported before [21]. The reaction rates were estimated and fitted with the Langmuir–Hinshelwood first-order kinetic model. The degradation rate (*r*) was calculated using Equation (1) [3,9,10]:

*r* = K × C_0_^n^, (1)

where K is the rate constant, C_0_ is the initial MC-LR concentration, and *n* is the order of the reaction. The MC-LR photodegradation efficiency (E %) using the as-prepared and calcined TiO_2_ samples under visible light was determined using Equation (2) [9,10]:

*E*% = (1 − (C/C_0_)) × 100, (2)

where C_0_ and C are the MC-LR concentrations before and after irradiation, respectively.

## 3. Results and Discussion

### 3.1. Characteristics of Photocatalyst

**Figure 1:**
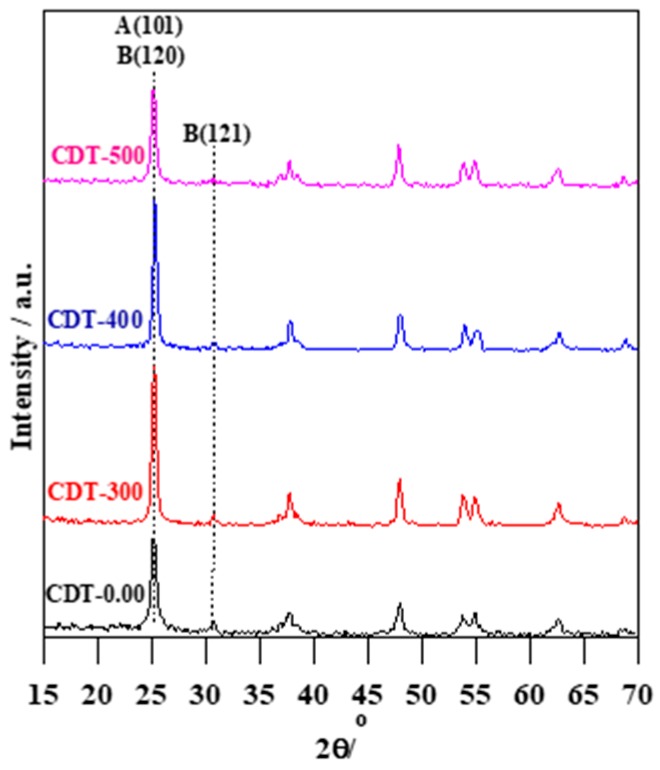
XRD patterns for CDT-0.00, CDT-300, CDT-400, and CDT-500 samples.

The crystallographic structure of TiO_2_ powders, obtained by thermal hydrolysis of an aqueous solution of Ti_2_(SO_4_)_3_, which was pre-oxidized by sodium nitrate in the presence of 1 mol L^−1^ glycine at pH 10 and then calcined at different temperatures (300, 400, and 500 °C), was investigated by XRD. The XRD patterns of CDT-0.00, CDT-300, CDT-400, and CDT-500 samples are displayed in Figure 1. The diffraction peaks of all samples showed mixed-phase titania of anatase and brookite. The peaks at 25.27° (101), 36.96° (103), 37.76° (004), 38.56° (112), 47.84° (200), 53.76° (105), 55.04° (121), 62.56° (204), and 68.64° (116) were well attributed to anatase-TiO_2_ (JCPDS No. 84-1286) [9,10]. Additionally, the formation of brookite TiO_2_ phase (JCPDS no.15-0875) could be confirmed by the peaks at 25.27° (120), 30.72° (121), 37.76° (201), 47.84° (231), 53.76° (320), 55.04° (241), and 68.64° (400) [9,10]. Although almost all brookite peaks overlapped with those of anatase, the most intensive brookite peak at 30.72° clearly indicated its presence. It should be pointed that brookite (metastable) is not a common titania polymorph, whereas anatase (metastable) and rutile (stable) have been frequently reported. Therefore, these results indicate the role of glycine, Na^+^, and pH value in the formation of anatase-brookite TiO_2_. It has been proposed that insoluble titanium hydroxide (Ti(OH)_4_) is firstly obtained by the direct hydrolysis of a mixture of aqueous Ti_2_(SO_4_)_3_ and NaNO_3_ [9,10,22]. Then, by adding sodium hydroxide (to adjust pH to be 10) and glycine, the insoluble sodium titanate (Na_2-X_H_2_Ti_2_O_5_.H_2_O) and soluble five-membered ring complex [Ti(OH)_x_(C_2_H_5_NO_2_)_y_]^z−^ are formed during the hydrothermal treatment [10,22,23,24,25]. The sodium titanate is transformed into brookite at a high temperature in highly alkaline medium, whereas [Ti(OH)_x_(C_2_H_5_NO_2_)_y_]^z−^ is transferred into anatase to obtain anatase-brookite heterojunction titania [10,22]. The content of the anatase and brookite phases were calculated using Zhang formula [26]. It was observed that an increase in calcination temperature resulted in a decrease in brookite content. Therefore, it was found that brookite was gradually converted to anatase over this temperature range (see Figure 1), and/or amorphous titania was transformed to anatase (as suggested by more intensive anatase peaks). Although some reports suggest the direct transition of brookite into rutile (not via anatase) [27] and an accelerated anatase to rutile transition (starting at 500 °C) in the presence of brookite [28], this study showed that the brookite presence does not necessarily cause fast and direct anatase-to-rutile transition (even at 500 °C). The Scherrer’s equation was applied to estimate the average crystallite size for the anatase and brookite phases from the broadening of peaks 25.27° (120) and 30.72° (121), respectively (see Table 1) [10]. Clearly, both anatase and brookite crystallite sizes showed a typical upward trend with increasing calcination temperature. The change in phase structure and particle size of TiO_2_ with increasing calcination temperature is corroborated by some previous reports [29,30,31]. For example, Allen et al. studied the influence of calcination temperature on the morphology (crystalline phase and size) and photocatalytic performance of TiO_2_. They noticed a similar trend for the calcination of anatase-brookite TiO_2_ samples, where anatase content increased gradually with increasing temperature, in contrast to the decrease in brookite content. Moreover, the crystal size of anatase and brookite increased with increasing calcination temperature [31].

**Figure 2:**
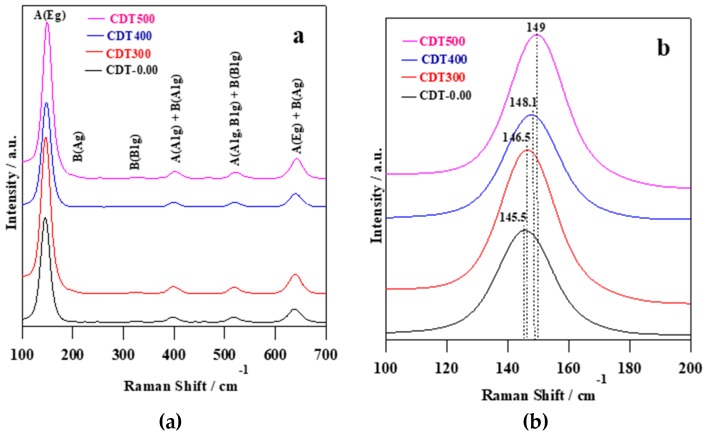
(**a**) Raman spectra of CDT-0.00, CDT-300, CDT-400, and CDT-500 samples; and (**b**) enlargement of the main peak around 145 cm^−1^.

To confirm the phase structure of the obtained TiO_2_, Raman spectroscopy was used. In Figure 2a and Table 2, it is explicit that all the TiO_2_ samples exhibited certain peaks matching to the vibration modes of anatase and brookite [9,32]. Figure 2b shows the main Raman peak of anatase (Eg mode, peak position at around 145 cm^−1^). The peak was red-shifted and its width increased with increasing temperature, and these observations are consistent with the literature data [33]. The redshift and broadening of Raman peaks of Eg mode can be assigned to thermal expansion, intrinsic anharmonicity and phonon confinement effects [33]. Heating results in expanding of material leading to redshift, and the intrinsic anharmonicity is stronger with temperature [33,34]. The phonon–phonon interactions, which may be due to the O-Ti-O bond vibrational type, were very obvious in the Eg mode, hence the phonon confinement effects should cause an increase in the shift and asymmetric broadening of Raman peaks [33,34].

**Figure 3:**
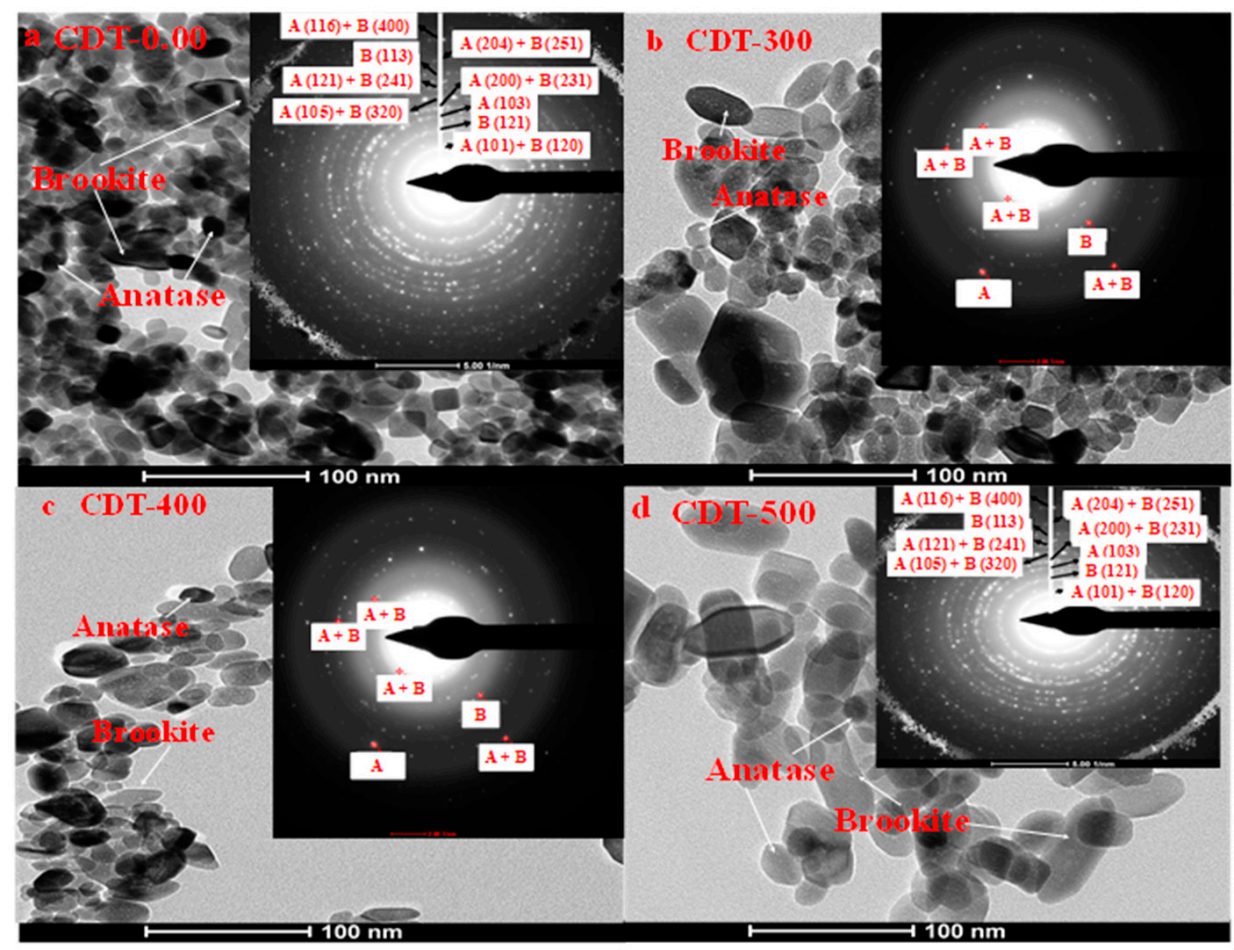
(**a**–**d**) TEM images of CDT-0.00, CDT-300, CDT-400, and CDT-500 samples, respectively.

Figure 3 shows exemplary TEM images and the corresponding electron diffraction (ED) patterns of all samples obtained at different calcination temperatures. All samples contained nano-rod-like particles and small nano-quasi-spherical-like particles. Since brookite has orthorhombic structure, in which TiO_6_ octahedron shares three edges and corners, the rod-like particles of brookite have frequently been reported [35,36,37,38,39]. Therefore, it was assumed that the small nanoparticles Were related to the anatase phase, whereas brookite formed the rod-like particles. Similar to crystallite size, the particle sizes increased with an increase in the calcination temperature. According to the insets in Figure 3a–d, the interface, as revealed by the electron diffraction (ED) patterns, consisted of anatase/brookite, being consistent with XRD data.

**Figure 4:**
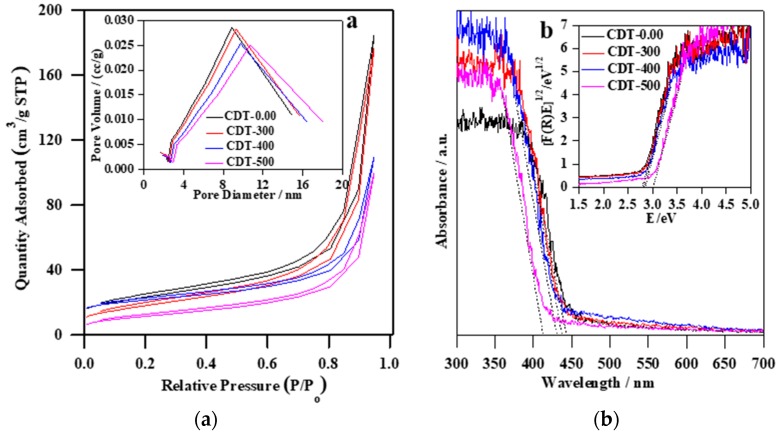
(**a**) N_2_ sorption isotherm and pore size distribution (inset); and (**b**) diffuse reflectance spectra and Tauc plots (inset) for all obtained samples.

Figure 4a shows the nitrogen adsorption–desorption isotherms and pore-size distribution (inset) of CDT-0.00, CDT-300, CDT-400, and CDT-500 samples. It was found that all TiO_2_ samples exhibited IV-type isotherm according to IUPAC classification, proving the formation of a mesoporous structure [32,40]. The broad hysteresis loops (0.35–0.96 P/P_o_) for CDT-0.00 and CDT-300 samples and narrow ones (0.53–0.96 P/P_o_) for CDT-400 and CDT-500 samples indicate that thermal treatment caused a shift for hysteresis loops to higher relative pressure, and this might correlate with the gradual loss of mesoporous structure with increasing temperature [41]. Moreover, it should be mentioned that, with increasing temperature, the particle and pore sizes of the TiO_2_ photocatalyst increased, resulting in narrowing the hysteresis loops because the large particles could be considered as irregular voids [42]. Additionally, it must be pointed that thermal treatment influenced all surface properties, e.g., pore sizes increased from 8.6 to 10.7 nm, whereas pore volume and specific surface area decreased from 0.29 to 0.23 cm^3^ g^−1^ and 72.4 to 20.5 m^2^ g^−1^, respectively, by thermal treatment at 500 °C (as summarized in Table 1). Figure 4b presents the diffuse reflectance spectra and band gap estimations (inset) of as-prepared and calcined TiO_2_ photocatalysts. The increase in calcination temperature resulted in the hypochromic effect, i.e., shift of the absorption edge towards shorter wavelengths, demonstrating the broadening of the band gap (from 2.79 to 3.00 eV), as shown in Figure 4b and Table 1. Generally, brookite shows broader bandgap than anatase, ca. 3.3 eV, but the modification resulted in bandgap narrowing; however, temperature again caused an increase in the band gap. Accordingly, it is proposed that, by applying the thermal treatment, the non-metal elements released gradually, hence the visible light response of the samples was diminished, resulting in redshift of absorption edge and broadening of band gap, similarly to previous reports [43,44].

**Figure 5:**
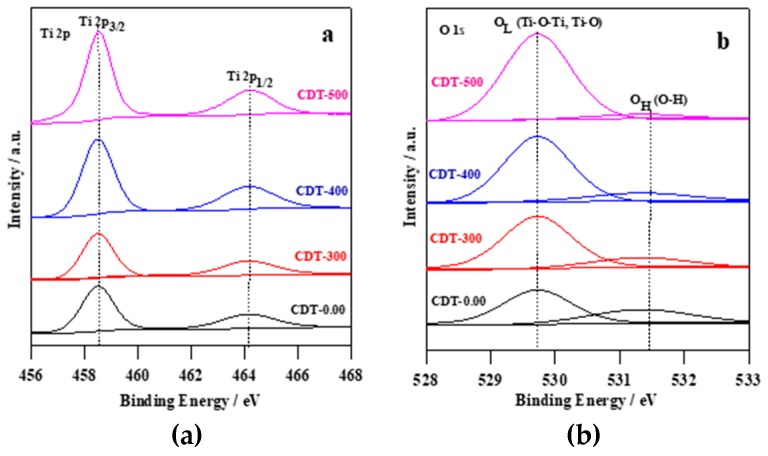
XPS detailed scans in the energy regions of: (**a**) Ti 2p; and (**b**) O 1s of all samples.

**Figure 6:**
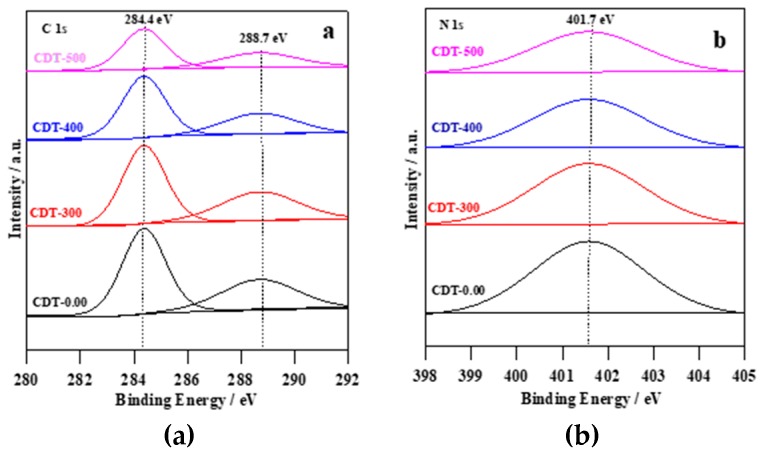
XPS detailed scans in the energy regions of: (**a**) C 1s; and (**b**) N 1s of the obtained photocatalysts.

**Figure 7:**
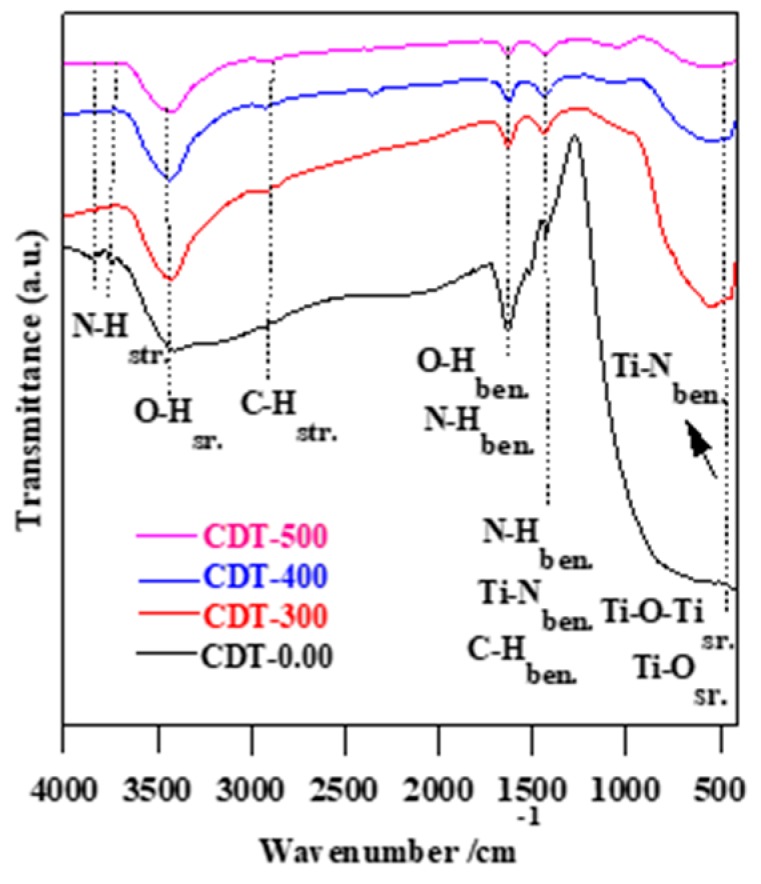
FT-IR of CDT-0.00, CDT-300, CDT-400, and CDT-500 samples.

To determine the nature of incorporated elements, the modified TiO_2_ photocatalysts were also characterized by XPS and FT-IR (see Figure 5, Figure 6 and Figure 7). Figure 5a shows XPS spectra of the samples for titanium (Ti 2p). All samples exhibited two Ti components with the core level binding energies at 458.5, and 464.2 eV for Ti 2p_3/2_ and Ti 2p_1/2_, respectively. These peaks are attributed to TiO_2_ lattice (Ti-O-Ti) [7,8,10,19,32]. Figure 5b gives the XPS of O 1s spectra of the TiO_2_ samples. The samples displayed two O components with the O 1s binding energies at 529.7 and 531.6 eV. The low binding energy is ascribed to oxygen in TiO_2_ lattice (Ti−O−Ti), and the higher one to the surface hydroxyl groups, resulting from chemisorbed water [7,8,10,19,32]. It was observed that the content of surface hydroxyl groups decreased with increasing the calcination temperature, and this is consistent with the FTIR results. The decrease in surface hydroxyl groups might lead to reducing the possibility for trapping photogenerated holes, and thus enhancing the possibility of the electron–hole recombination, which agrees with the PL results [7,8,10,19,32,45]. The XPS spectra for C 1s are presented in Figure 6a. Two peaks were observed with the binding energies of 284.4 and 288.7 eV, which were assigned to C-C, and C-H and C-O, C=O, O=C-O, Ti-O-C, and C-N bonds, respectively [10,19,32]. For nitrogen (N 1s), one peak with binding energy of 401.7 eV might correspond to the chemisorbed N species (NO, N_2_O, NO^2-^, and NO^3−^), hyponitrite species, interstitial N-doping (Ti-O-N and Ti-N-O linkage) and substitutional N-doping (O-Ti-N linkage) (see Figure 6b) [10,19,32]. The surface compositions of C/N co-modified TiO_2_ samples are summarized in Table 3. It indicates that the atomic concentration of Ti and O increased with increasing temperature, while O/Ti ratio remained almost unchanged. On the other hand, the content of N and C decreased with increasing calcination temperature, which might be explained by the thermal release of surface modifiers (non-metal elements), as already reported [43,44].

The existence of carbon and nitrogen for the obtained TiO_2_ powders was further confirmed by FTIR spectroscopy, as shown in Figure 7. It was found that prepared TiO_2_ samples displayed vibration modes at the 4000–400 cm^−^^1^ range, indicating the possible incorporation of C and N in the TiO_2_ lattice (see Figure 7 and Table 4) [9,10,32,40]. The Ti–O bond and Ti–O–Ti bridge stretching are located at 900–400 cm^−^^1^ [9,10]. The low FTIR bands located at 480 cm^−^^1^ are attributed to Ti-N_str._ bonds [10,32]. The bands located at 1416 cm^−^^1^ are related to Ti-N_ben._, C-H_ben._, and N-H_ben._ [10,32]. N-H_ben._ and O-H_str._ bonds are located at 1630 cm^−^^1^ [9,10,32,40]. The FTIR bands located at 2922 cm^−^^1^ are assigned to C-H_str._ [10]. The broad bands located at 3418 cm^−^^1^ are characterized to O-H_str._ [9,10,32,40]. The weak FTIR bands located at 3700–3800 cm^−^^1^ are due to N-H_str_. [10,32]. It is worth noting that the intensities of O-H, N-H, C-H and Ti-N vibration bands decreased with increasing calcination temperature, confirming XPS data, i.e., the thermal treatment caused a release of non-metal elements and/or chemosorbed H_2_O [46,47,48]. 

### 3.2. Photocatalytic Activity of Photocatalyst

#### 3.2.1. Degradation of MC-LR over TiO_2_ Photocatalyst

**Figure 8:**
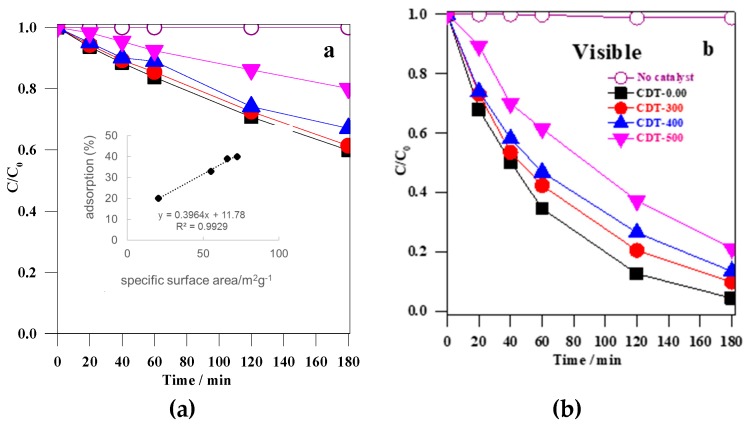
Change of the MC-LR concentration with initial concentration 10 mg L^−1^ versus reaction time: (**a**) under dark condition; and (**b**) under visible light illumination for 3 h using CDT-0.00, CDT-300, CDT-400, CDT-500 catalysts (0.3 g L^−1^) at pH 6.3.

**Figure 9:**
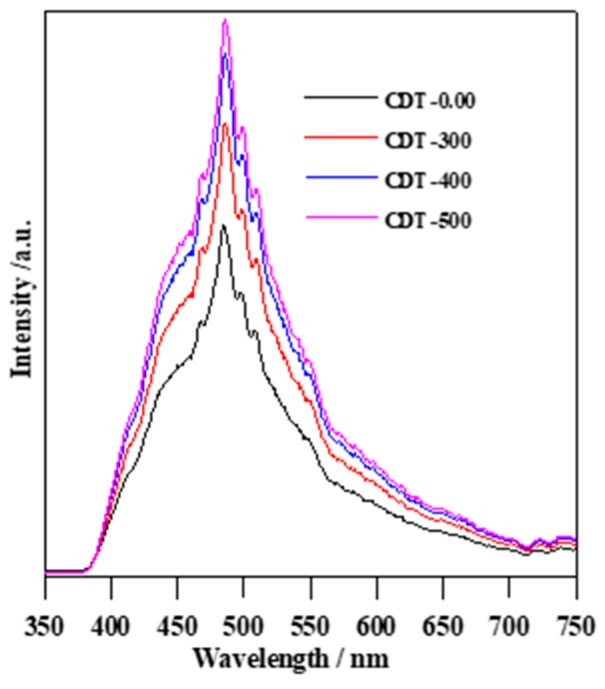
PL spectra of CDT-0.00, CDT-300, CDT-400, and CDT-500 catalysts.

To compare the catalytic activity of as-synthesized (CDT-0.00) and calcined (CDT-300, CDT-400, and CDT-500) C-N co-modified TiO_2_ for MC-LR removal, three kinds of experiments were performed: MC-LR adsorption in the dark (blank test), MC-LR photolysis (without photocatalyst), and MC-LR photodegradation (visible light + photocatalyst). In the initial experiments, the pH value and the content of TiO_2_ were 6.3 and 0.3 g L^−1^, respectively. Before the photocatalytic reaction, the adsorption of MC-LR on the surface of co-modified TiO_2_ samples in the dark was performed (see Figure 8a). It was found that 3-h stirring resulted in 40%, 39%, 33% and 20% adsorption of MC-LR on the surface of CDT-0.00, CDT-300, CDT-400 and CDT-500, respectively. More efficient adsorption of MC-LR on the surface of as-synthesized co-modified TiO_2_ than that on the calcined samples could result from larger specific surface area, as shown in inset of Figure 8a. Blank tests (in the absence of photocatalyst and irradiation) indicated that MC-LR is stable and irradiation was necessary for its efficient decomposition (Figure 8a,b). It was found that 3-h vis irradiation resulted in almost complete degradation of MC-LR, reaching 95% (0.0172 mni^−1^, r = 1.04 × 10^−4^ mmol L^−1^), 90% (0.0131 mni^−1^, r = 0.81 × 10^−4^ mmol L^−1^), 86% (0.0113 min^−1^, r = 0.76 × 10^−4^ mmol L^−1^), and 79% (0.0084 mn^−1^, r = 0.68 × 10^−4^ mmol L^−1^) removal efficiencies for CDT-0.00, CDT-300, CDT-400, and CDT-500 samples, respectively, as shown in Figure 8b. Therefore, it was confirmed that all co-modified titania samples displayed a high photocatalytic performance. Several reasons could be responsible for this high photocatalytic activity, such as formation of mixed-phase structure (anatase/brookite (A/B)), mesoporous morphology, and non-metal co-modification [9,10,11,12,13,14,15,16,17,18,19,22,25,32,48]. The formation of A/B mixed-phase TiO_2_ might facilitate the migration of electrons from brookite to anatase, and holes from anatase to brookite, and hence reducing the possibility of electron–hole recombination [9,10,22,25]. The high photocatalytic activity of the mesoporous TiO_2_ can be explicated by: (i) more active sites for photocatalytic reaction; (ii) the cumulation of hydroxyl radicals inside the pores; (iii) the high dispersion of mesoporous TiO_2_ in the aqueous solution; and (iv) the rapid diffusion of MC-LR to the reactive sites on the surface of the mesoporous TiO_2_ photocatalyst [49,50]. The high activity of the photocatalysts under visible light can be attributed to narrowing of band gap resulting from non-metal modification, as shown in our previous report on non-modified titania [32]. Moreover, it was observed that the degradation rate was decreased with an increase in calcination temperature, which might be explained by: (i) the increases in crystal and pore sizes; and (ii) the decreases in specific surface area and the content of non-metal elements. It is worth noting that the charge-carriers recombination was gradually increasing with increasing the calcination temperature, probably due to a decrease in the amount of adsorbed water molecules, which play an essential role in the photocatalytic activity since they can react with photogenerated holes forming reactive oxygen species (ROS), thereby inhibiting charge recombination [19]. These hypotheses were confirmed by changes in the XPS spectra (a decrease in hydroxyl groups), FTIR bands (O-H vibration) and PL intensities (Figure 5, Figure 7 and Figure 9, respectively) with an increase in calcination temperature. For example, the intensity of the bands located at 3418 and 1630 cm^−1^, attributed to the O-H vibration modes of adsorbed water molecules, decreased with increasing the calcination temperature (Figure 7). 

The photoluminescence (PL) spectroscopy has been used widely to investigate the charge separation/recombination of photogenerated charges. Additionally, the PL emission intensity is related directly to the recombination rate of the e^−^/h^+^ pairs, i.e., the lower is the PL emission intensity, the lower is the recombination rate, hence an increase in the photocatalytic activity of the materials [10,33]. Indeed, higher PL intensity of the sample CDT-0.00 than those of calcined samples (Figure 9) correlates well with photocatalytic activity (Figure 8), confirming the lower charge carriers’ recombination. The results indicate that PL peaks of all samples are located at the same position around 485 nm, which can be attributed to the free electron recombination process from the conduction band (CB) to the ground state recombination center [32]. The PL intensities increased with increasing the calcination temperature, i.e., the as-synthesized TiO_2_ catalyst (CDT-0.00) exhibited the lowest PL intensity indicating the lowest charge carriers’ recombination, and thus the highest photocatalytic activity for MC-LR degradation.

#### 3.2.2. Improvement of Photocatalytic Degradation 

**Figure 10:**
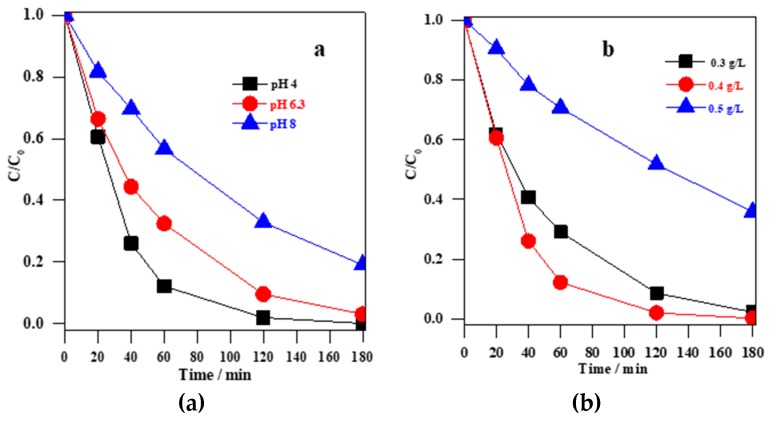
(**a**) Photodegradation of 10 mg L^−1^ MC-LR in the presence of CDT-0.00 (0.4 g L^−1^) under visible irradiation at different pH values (4, 6.3, and 8); and (**b**) photodegradation of 10 mg L^−1^ MC-LR using different dosages of CDT-0.00 (0.3, 0.4, and 0.5 g L^−1^) under visible irradiation at pH 4.

To attain the highest efficiency of MC-LR degradation on co-modified TiO_2_ under vis irradiation, the condition-dependent activity was investigated, i.e., the influence of the initial pH value, the initial content of TiO_2_, and the initial concentration of MC-LR. Firstly, the pH effect on the degradation of MC-LR (10 mg L^−1^) on CDT-0.00 photocatalyst (0.4 g L^−1^) was investigated for three different pH values (4, 6.3, and 8), and the data are shown in Figure 10a. The complete MC-LR degradation (100%, K = 0.034 min^−1^, and r = 2.01 × 10^−4^ mmol L^−1^) was achieved at the acidic pH 4, whereas at more neutral (pH 6.3) and basic (pH 8) conditions, the total degradation efficiencies were lower reaching 97% (K = 0.019 min^−1^, and r = 1.16 × 10^−4^ mmol L^−1^) and 81% (K = 0.009 min^−1^, and r = 0.59 × 10^−4^ mmol L^−1^), respectively. The enhanced photocatalytic degradation of MC-LR in acidic conditions has already been reported [3,4]. It is well known that the TiO_2_ surface is positively/negatively charged below/above the point of zero charge (p_zc_ = 6–8 depending on titania sample) [51,52,53,54,55]. Hence, the TiO_2_ surface is positively charged (Ti-OH_2_^+^) at pH 4. It is important to note that MC-LR is negatively charged above pH 2.10, and positively charged below this value [56]. Therefore, obtained results, i.e., best activity at pH 4, suggest that attractive forces between the positively charged TiO_2_ (TiOH_2_^+^) and the negatively charged MC-LR (MC-LRH^-^) enhance the adsorption of MC-LR on the surface of C/N co-modified TiO_2_ photocatalyst, thus improving its photocatalytic degradation. 

Next, the influence of photocatalyst dose was investigated, and obtained data are shown in Figure 10b for three different values (0.3, 0.4, and 0.5 g L^−1^) at acidic conditions (pH 4) and for initial concentration of MC-LR of 10 mg L^−1^. It was found that the photocatalytic efficiency increased with an increase in the photocatalyst content from 0.3 to 0.4 g L^−1^ (from 98% (K = 0.021 min^−1^, and r = 1.27 × 10^−4^ mmol L^−1^) to 100% (K = 0.034 min^−1^, and r = 2.01 × 10^−4^ mmol L^−1^)), due to more efficient photon absorption, similar to reported data [21,39,57,58]. However, further increase in the photocatalyst dose (0.5 g L^−1^) resulted in a decrease in the photocatalytic efficiency to 64% (K = 0.006 min^−1^, and r = 0.37 × 10^−4^ mmol L^−1^), due to the “inner filter” effect (reduced penetration depth and increased scattering of the incident light beam) [20,56]. Therefore, for reliable comparison between different photocatalysts the photoirradiation, experiments should be performed at maximum photon absorption, i.e., after reaching plateau in photocatalyst dose-activity dependence, as clearly discussed by Kisch [58]. Although, usually for titania powders of specific surface areas of about 50–200 m^2^g^−1^, the optimum dose in the range of 0.5–3.0 g L^−1^ [58] has been proposed, our data indicate that even 0.5 g L^−1^ could be too large (probably due to intensive coloration by C/N-modification).

**Figure 11:**
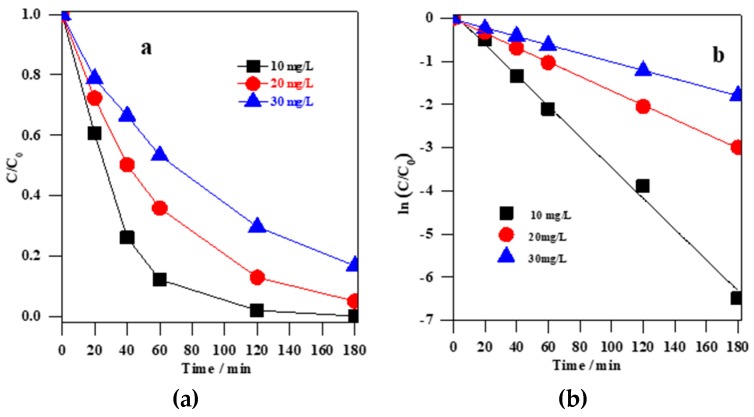
(**a**) Photodegradation of MC-LR with different initial concentration (10, 20, and 30 mg L^−1^), using 0.4 g L^−1^ CDT-0.00 under visible irradiation at pH 4; and (**b**) plot of C/C_0_ vs irradiation time.

The influence of initial MC-LR concentration on the photocatalytic performance of as-synthesized C/N co-modified TiO_2_ with an initial TiO_2_ dose of 0.4 g L^−1^ at pH 4 is shown in Figure 11. It was found that an increase in MC-LR concentration resulted in a decrease in degradation efficiency. This obvious behavior is caused by the fact that higher concentration means higher competition (more molecules) for same content of reactive oxygen species, as well as the competition between oxidation by-products and original compound (MC-LR) for both adsorption (on the photocatalyst surface) and degradation. The MC-LR degradation presented a first-order behavior (as confirmed by logarithmic plot (Figure 11b)) with the reaction rates of 2.01 × 10^−4^ mmol L^−1^ (K = 0.034 min^−1^), 1.03 × 10^−4^ mmol L^−1^ (K = 0.017 min^−1^), and 0.73 × 10^−4^ mmol L^−1^ (K = 0.011 min^−1^) and degradation efficiency after 180-min vis irradiation of 100%, 95% and 83% at the initial MC-LR concentration of 10, 20, and 30 mg L^−1^, respectively.

#### 3.2.3. Proposed Mechanism for MC-LR Removal 

##### (i) Adsorption:

**Scheme 1:**
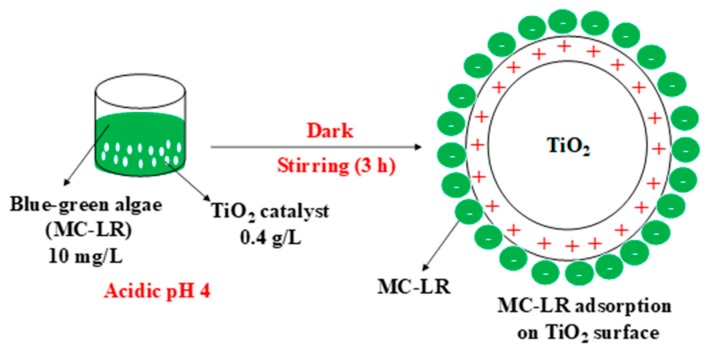
Proposed mechanism of MC-LR adsorption on co-modified TiO_2_ at acidic pH 4.

Before irradiation, an aqueous solution of MC-LR was stirred with C/N co-modified TiO_2_ powder in the dark for 180 min to attain the adsorption equilibrium for MC-LR on the photocatalyst surface. Scheme 1 shows the proposed mechanism of MC-LR adsorption on the TiO_2_ surface in the dark at acidic conditions, i.e., fast adsorption due to the attractive forces between the positively charged TiO_2_ (TiOH_2_^+^) and the negatively charged MC-LR (MC-LRH^-^).

##### (ii) Photodegradation:

**Scheme 2:**
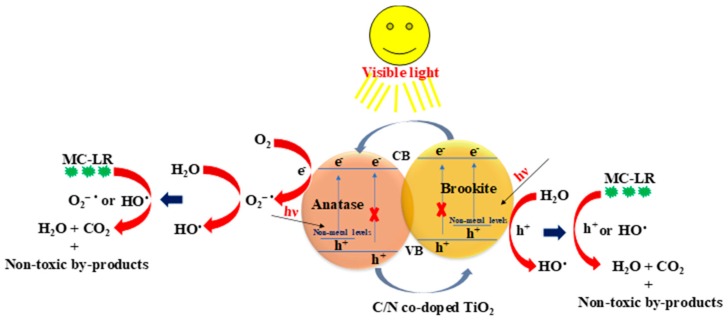
Possible mechanism of MC-LR degradation over C/N co-modified A/B TiO_2_ photocatalyst.

The proposed mechanism of the MC-LR photodegradation on C/N co-modified TiO_2_ photocatalyst is shown in Scheme 2. Firstly, vis irradiation could excite semiconductor only from the impurity energy levels (non-metal levels, considering surface modification with doping-like nature, e.g., surface oxygen replaced by nitrogen and carbon) to the conduction band, and leaving photo-generated holes at the impurity levels in both anatase and brookite phase, as presented in Equation (3).

(3) $${\mathrm{TiO}}_{2}+h\nu \;\overset{Photo-excitation}{\to }\;h^{+}+e^{-},$$

Moreover, it is possible (and has already been proposed in our previous and other reports [9,10,22,25,32,40,47,48]) that photogenerated electrons could transfer from brookite to anatase, whereas the holes could migrate inversely (from anatase to brookite), thereby enhancing the electron–hole separation. Following that, these separated charges can oxidize and reduce the adsorbed H_2_O and O_2_, respectively, to form high reactive oxygen species (ROS) by the following reactions:

(4) $$H_{2}O+h^{+}\;\overset{Oxidation}{\to }\;{}^{•}\mathrm{OH},$$

(5) $$O_{2}+e^{-}\;\overset{Reduction}{\to }\;{}^{•-}O_{2},$$

(6) $${}^{•-}O_{2}+H_{2}O\;\vec{\;\;}{\;}^{•}\mathrm{OH}$$

Finally, MC-LR adsorbed on the TiO_2_ surface can be degraded either directly by h^+^ or indirectly by formed ROS (Equations (4)–(6)) to be converted finally into non-toxic products, H_2_O and CO_2_, as shown in Equation (7). 

(7) $$\mathrm{MC}\text{-}\mathrm{LR}+h^{+},{\;}^{•}\mathrm{OH},\;(\mathrm{and}/\mathrm{or})\;{}^{•-}O_{2}\;\overset{Degradation}{\to }\;H_{2}O+{\mathrm{CO}}_{2}+\mathrm{non}\text{-}\mathrm{toxic}\;\mathrm{by}\text{-}\mathrm{products}$$

## 4. Summary and Conclusions

MC-LR toxic pollutant was removed from aqueous solution over visible light responsive C/N co-modified, mesoporous, mixed-phase (A/B) TiO_2_ photocatalyst, synthesized through one-pot hydrothermal approach using glycine as C/N source. The as-synthesized and calcined TiO_2_ at different temperatures (300, 400, and 500 °C) were prepared, and characterized by XRD, Raman, TEM, BET-surface area, UV-vis-DRS, XPS, FTIR, and PL. The important parameters, including the initial pH value, the photocatalyst content, and MC-LR concentration, were studied to reach the best conditions for photocatalytic degradation of MC-LR using the co-modified TiO_2_ under visible irradiation. The results reveal that all mesoporous co-modified A/B TiO_2_ photocatalysts exhibited high photocatalytic activity toward MC-LR degradation, probably because of their mesoporous structure and non-metal modification. The photocatalytic efficiency decreased with increasing calcination temperature, as a result of decreased specific surface area and a release of non-metal modifiers. The complete degradation of 10 mg L^−1^ MC-LR over 0.4 g L^−1^ as-synthesized C/N co-modified TiO_2_ at pH 4 was achieved under vis irradiation for 180 min. It is proposed that mixed-phase formation and non-metal co-modification cause the high photocatalytic activity under visible light irradiation, as shown in Scheme 2. Therefore, it is concluded that this photocatalyst is suitable for efficient water/wastewater treatment under natural solar radiation.

## Figures and Tables

**Table 1:** Crystal size, BET surface area, pore volume, pore size and band gap of CDT-0.00, CDT-300, CDT-400, and CDT-500 samples.

Samples	Crystal Size (nm)		BET Surface Area (m^2^ g^−1^)	Pore Volume (cm^3^ g^−1^)	Pore Size (nm)	Band Gap (eV)
	A	B				
CDT-0.00	16	32	72.41	0.29	8.6	2.79
CDT-300	17	35	66.00	0.27	9.3	2.82
CDT-400	20	42	55.22	0.26	9.8	2.86
CDT-500	25	50	20.51	0.23	10.7	3.00

**Table 2:** Raman vibration bands and their assignments in CDT-0.00, CDT-300, CDT-400, and CDT-500 samples at 532 nm excitation wavelength.

Samples	Assignment [Peak Position (cm^−1^)]	
	Anatase	Brookite
CDT-0.00	Eg [145.5, 636], B1g [517.5], A1g [396, 517.5]	Ag [199, 636], A1g [396], B1g [517.5]
CDT-300	Eg [146.5, 639], B1g [518], A1g [397, 518]	Ag [199, 639], A1g [397], B1g [323, 518]
CDT-400	Eg [148.1, 641.5], B1g [518.1], A1g [399, 518.1]	Ag [199, 641.5], A1g [399], B1g [518.1]
CDT-500	Eg [149, 644], B1g [521], A1g [400, 521]	Ag [199, 644], A1g [400], B1g [323, 521]

**Table 3:** The surface composition of Ti, O, C, and N obtained from XPS.

Samples	Content (at. %)				Ratio
	Ti 2p	O 1s	C 1s	N 1s	O/Ti
CDT-0.00	20.20	56.20	21.70	1.90	2.8
CDT-300	20.64	57.60	20.10	1.66	2.8
CDT-400	21.05	58.10	19.40	1.45	2.8
CDT-500	21.98	58.60	18.20	1.22	2.7

**Table 4:** FTIR vibration bands and their locations for CDT-0.00, CDT-300, CDT-400, and CDT-500 samples.

Vibration Mode	Bands Location (cm^−1^)
Ti-O; Ti-O-Ti (stretching)	400–900
O-H (stretching)	3418
O-H (bending)	1630
N-H (stretching)	3700–3800
N-H (bending)	1630; 1416
C-H (stretching)	2922
C-H (bending)	1416
Ti-N (bending)	1416, 480
